# Comparison of the Hematotoxicity of PRRT with Lutathera^®^ and Locally Manufactured ^177^Lu-HA-DOTATATE in Patients with Neuroendocrine Tumors and the Impact of Different Application Intervals

**DOI:** 10.3390/cancers17091423

**Published:** 2025-04-24

**Authors:** Markus Hofmann, Sophie C. Kunte, Marcus Unterrainer, Astrid Delker, Adrien Holzgreve, Johannes Toms, Franz Joseph Gildehaus, Christoph J. Auernhammer, Christine Spitzweg, Mathias J. Zacherl, Harun Ilhan, Johannes Rübenthaler, Leonie Beyer, Lena M. Unterrainer

**Affiliations:** 1 Department of Nuclear Medicine, LMU University Hospital, LMU Munich, 81377 Munich, Germany; sophie.kunte@med.uni-muenchen.de (S.C.K.); marcus.unterrainer@die-radiologie.de (M.U.); astrid.delker@med.uni-muenchen.de (A.D.); johannes.toms@med.uni-muenchen.de (J.T.); franz.gildehaus@med.uni-muenchen.de (F.J.G.); mathias.zacherl@med.uni-muenchen.de (M.J.Z.); harun.ilhan@die-radiologie.de (H.I.); leonie@beyerfamilie.de (L.B.); lena.unterrainer@med.uni-muenchen.de (L.M.U.); 2 Bavarian Cancer Research Center (BZKF), Partner Site Munich, 80539 Munich, Germany; 3 Die Radiologie, 80331 Munich, Germany; 4 Department of Nuclear Medicine, David Geffen School of Medicine, UCLA, Los Angeles, CA 90095, USA; 5 ENETS Centre of Excellence, Interdisciplinary Center of Neuroendocrine Tumours of the GastroEnteroPancreatic System (GEPNET-KUM), LMU University Hospital, LMU Munich, 81377 Munich, Germany; christoph.auernhammer@med.uni-muenchen.de (C.J.A.); christine.spitzweg@med.uni-muenchen.de (C.S.); 6 Department of Internal Medicine 4, LMU University Hospital, LMU Munich, 81377 Munich, Germany; 7 Department of Radiology, LMU University Hospital, LMU Munich, 81377 Munich, Germany; johannes.ruebenthaler@med.uni-muenchen.de

**Keywords:** NET, PRRT, ^177^Lu-HA-DOTATATE, Lutathera^®^, hematotoxicity

## Abstract

Background/Objectives: Peptide Receptor Radionuclide Therapy (PRRT) is approved for patients with inoperable, progressive and/or metastatic well-differentiated NETs. Before the approval of Lutathera^®^, locally manufactured ^177^Lu-HA-DOTATATE was used on a regular basis in clinical routine. The aim of this study was (1) to compare the hematotoxicity of locally manufactured ^177^Lu-HA-DOTATATE with Lutathera^®^ in GEP-NET patients and (2) to compare the recommended treatment interval of 8 weeks between each cycle to a prolonged scheme of up to 11 weeks for both ^177^Lu-HA-DOTATATE and Lutathera^®^. Methods: The included patients with GEP NETs (*n* = 46) received four cycles of PRRT, either ^177^Lu-HA-DOTATATE or Lutathera^®^, and were divided into four subgroups. The subgroups were treated with either locally manufactured ^177^Lu-HA-DOTATATE or Lutathera^®^ and were stratified into a mean application interval of 8 (HA_8weeks_, *n* = 10/Lutathera_8weeks_, *n* = 16) or 11 weeks (HA_adapted_, *n* = 10/Lutathera_adapted_, *n* = 10). To evaluate therapy associated hemato- and nephrotoxicity, patients underwent two laboratory follow-up examinations (follow-up 1—between 2./3. therapy cycle; follow-up 2—after the termination of the 4. therapy cycle) and were then compared to pre-PRRT laboratory results. To assess hematological and renal recovery trends, blood values and parameters of kidney function were collected up to 58.9 weeks after PRRT completion. Common Terminology Criteria for Adverse Events version 5.0 (CTCAE v5.0) were used for grading hematological parameters. Results: The occurrence of high-grade adverse events (CTCAE grade 3/4) after PRRT was moderate, with 1/10 (10%) grade 4 lymphocytopenia in the Lutathera_adapted_ group, while overall, 20/46 (43.5%) patients had grade 3 lymphocytopenia. Grade 3 thrombocytopenia occurred in 1/10 (10%) patients of the HA_adapted_ group. Absolute and percentage changes in the kidney function (creatinine, TER) remained constant during PRRT in all subgroups. All four subgroups showed a significant decrease in absolute blood value changes for PLT counts, WBC counts, neutrophil granulocytes and lymphocytes between, prior to and after PRRT (*p* < 0.05, each). Regarding percentage changes in laboratory parameters, only the HA_adapted_ and the HA_8weeks_ groups had significant decreases in WBC (*p* < 0.03, each) and PLT counts (*p* < 0.04, each) while there was no significant degradation of any other hematological parameter in any of the subgroups. Only patients with longer treatment intervals under ^177^Lu-HA-DOTATATE therapy showed a statistically significant correlation in the long-term recovery analysis concerning the PLT counts (r = 0.6, *p* < 0.0001). Other blood and kidney values showed no significant correlation in the long-term analysis in the other subgroups. Conclusion: Comparing the hematotoxicity in patients that were treated with locally manufactured ^177^Lu-HA-DOTATATE with patients that were treated with Lutathera^®^ and assessing different treatment intervals in both groups (8 vs. 11 weeks), revealed that there is overall a low to moderate incidence of significant changes in hematological and renal parameters directly after PRRT. Recovery trends of hematological and renal parameters after 1 year suggest that patients treated with locally manufactured ^177^Lu-HA-DOTATATE might benefit from a longer treatment interval of 11 weeks regarding their PLT counts. Given the risk of developing hematological diseases such as therapy-related myeloid neoplasms years after PRRT, longer observational periods after PRRT will be crucial.

## Simple Summary

This study investigates the hematotoxicity associated with Peptide Receptor Radionuclide Therapy (PRRT) in patients with neuroendocrine tumors, focusing on a comparison between Lutathera^®^ and locally manufactured ^177^Lu-HA-DOTATATE and the influence of different application intervals. Since hematotoxicity remains a significant side effect of PRRT, the aim of this study is to provide crucial insights for clinical practice, aiming to optimize therapeutic strategies and tailor treatments for individual patients.

## 1. Introduction

Neuroendocrine tumors (NETs) are a rare heterogenous group of neoplasms and vary in biological characteristics and clinical presentation [1]. Around 62–67% derive from the gastrointestinal system and together they form the group of gastro-entero-pancreatic neuroendocrine tumors (GEP-NETs) [1]. By the time of initial diagnosis, roughly 20% of patients have distant metastases, which has a significant impact on survival [1,2]. According to the German and European consensus guidelines, PRRT with ^177^Lu-radiolabeled octreotide derivatives is a safe and effective form of targeted therapy for patients with inoperable and/or metastatic well-differentiated NETs with sufficient tumor uptake shown in diagnostic somatostatin receptor imaging [3]. Currently, PRRT with ^177^Lu-DOTATATE has the highest level of evidence as a therapeutic radiopharmaceutical for GEP NETs [3,4,5]. The prospective Phase 3 trial NETTER-1 revealed a longer PFS and OS in midgut NET patients when treated with the combination of ^177^Lu-DOTATATE and 30 mg octreotide long-acting release (LAR) compared to 60 mg octreotide LAR alone [6]. Subsequently, based on these findings, the first radiopharmaceutical for PRRT Lutathera^®^ was approved by the European Medicines Agency (EMA) in 2017 and by the U.S. Food and Drug Administration (FDA) in 2018 [7]. Lutathera^®^ has an indication for the treatment of inoperable or metastatic, progressive, well-differentiated (G1/G2), somatostatin receptor-positive GEP-NETs in adults. For Lutathera^®^, a treatment scheme of four infusions of 7.400 MBq each in an interval of 8 ± 1 weeks between each administration is recommended. However, the time interval can be extended up to 16 weeks in case of dose modifying toxicity [8,9]. Before the approval of Lutathera^®^, radiopharmaceuticals, such as ^177^Lu-HA-DOTATATE, used in PRRT were locally manufactured in nuclear medicine departments, leading to a limited availability of these products for most patients [10]. As the high-affinity form of DOTATATE, HA-DOTATATE has a slightly higher affinity for SSTR-2 and -5 than DOTATATE [11]. Locally manufactured ^177^Lu-HA-DOTATATE is still frequently used in some centers in a compassionate use program, also for other diseases than GEP NETs that overexpress somatostatin receptors, such as lung carcinoids, pheochromocytomas/paragangliomas (PPGLs) and meningiomas. From a radiochemical point of view, there are no substantial differences between the locally produced ^177^Lu-HA-DOTATATE and Lutathera^®^.

Dose-limiting organs of PRRT are kidneys and bone marrow with myelosuppression as the most common side effect. Therefore, blood values and parameters of kidney function need to be monitored regularly during and after PRRT [12,13]. Previous studies revealed that the incidence of subacute hematotoxicity after PRRT with ^177^Lu-DOTATATE is roughly 10% and incidences of severe hematotoxicity are low when tight screening and monitoring processes are applied [14]. Since there is no molecular biomarker predicting PRRT hematotoxicity, the protection of bone marrow reserve and kidney function is of great interest. Therefore, different strategies of myelo- and nephroprotection were evaluated in previous studies [15,16,17]. However, different adaptations of the time interval between each PRRT cycle were not yet the focus of preceding toxicity studies of PRRT.

The aim of this study was to compare the hematotoxicity of locally manufactured ^177^Lu-HA-DOTATATE with Lutathera^®^ in GEP-NET patients. In addition, application of PRRT in the advised treatment regimen of 8 weeks between each cycle was compared to a prolonged adapted scheme of up to 11 weeks. To evaluate hematological and renal recovery trends between the four subgroups, blood values and parameters of kidney function were collected after PRRT completion.

## 2. Materials and Methods

### 2.1. Patient Enrollment

The included patients received four cycles of PRRT, either ^177^Lu-HA-DOTATATE or Lutathera^®^, at the department of Nuclear Medicine, LMU University Hospital Munich. Patient selection for these therapies was based on inclusion criteria stated in current guidelines [13,18]. GEP-NET patients were divided into 4 therapy subgroups: 2 subgroups treated with locally manufactured ^177^Lu-HA-DOTATATE PRRT, 1 group with mean application intervals of 8 weeks (SD ± 0.1) (HA_8 weeks_, *n* = 10) between each cycle and the other with mean intervals of 11 weeks (SD ± 0.2) (HA_adapted_, *n* = 10). The other 2 subgroups were treated with Lutathera^®^ and were also separated by application intervals of 8 weeks (SD ± 0.1) (Lutathera_8 weeks_, *n* = 16) and 11 weeks (SD ± 0.1) (Lutathera_adapted_, *n* = 10). Thus, the term “adapted” refers to the two subgroups whose treatment interval was extended to 11 weeks (Lutathera_adapted_, HA_adapted_), compared to the two subgroups whose treatment interval was 8 weeks (Lutathera_8weeks_, HA_8weeks_). Sufficient tumor uptake was analyzed using somatostatin receptor imaging (^68^Ga-DOTATATE or ^18^F-SiTATE positron-emission-tomography combined with computed tomography (PET/CT)) prior to PRRT. This registry study was performed in compliance with the principles of the Declaration of Helsinki and its subsequent amendments, and with the approval of the local ethics committee (approval number 21-0102).

### 2.2. Radiopeptides

Radiolabeling of ^177^Lu-HA-DOTATATE was performed as stated in a previously described protocol, with slight modifications [19]. In the production process of ^177^Lu-HA-DOTATATE, non-carrier added ^177^Lutetium (EndolucinBeta^®^) was obtained from Isotope Technologies Munich S.E (Garching, Germany). DOTA-3-iodo-Tyr3-octreotate in GMP quality was provided by SCINTOMICS Molecular, Applied Theranostics Technologies GmbH (Fürstenfeldbruck, Germany). The precursor HA-DOTATATE (80 nmol or 125 μg) dissolved in 0.4 M sodium-acetate buffer (pH 4.5, 1.5 mL) was directly added to the ^177^Lu-vial (7.6 GBq ^177^Lu in approx. 200 µL 0.04 M HCl) and the mixture was heated for 20 min at 95 °C. The labeled product was directly diluted with 9 mL WFI (B.Braun, Melsungen, Germany) without further purification steps. The resulting solution was passed through a 0.22-μm filter into a sterile injection vial and dispensed for injection. A sample was taken for determination of identity, radiochemical purity, pH, apyrogenicity and sterility (after decay).

Lutathera^®^ was obtained commercially from Advanced Accelerator Applications, a Novartis Company (Colleretto Giacosa, Italy).

### 2.3. ^177^Lu-HA-DOTATATE Treatment

Biotherapy with somatostatin analogs (SSA) was paused at least 28 days prior to each treatment cycle. For nephroprotection, co-infusion of positively charged amino acids (2.5% Lysine and 2.5% Arginine) was started 30 min before each cycle. ^177^Lu-HA-DOTATATE and Lutathera^®^ were injected intravenously within 30 ± 10 min according to previous published injection recommendations.

### 2.4. Evaluation of Toxicity

Pre-PRRT laboratory analyses were performed one day prior to each treatment cycle. To evaluate any therapy associated hemato- or nephrotoxicity, patients underwent two follow-up examinations: follow-up 1 was performed in between the second and third therapy cycle and follow-up 2 after the termination of the fourth PRRT cycle. In both the Lutathera_8weeks_ and HA_8weeks_ group, follow-up 1 was performed after a mean time of 15 ± 1 weeks after the first PRRT cycle and follow-up 2 was performed after a mean time of 19 ± 0.4 weeks after follow-up 1. Patients of the Lutathera_adapted_ and the HA_adapted_ group had follow-up 1 after a mean time of 19 ± 0.5 weeks after PRRT initiation and follow-up 2 after a mean time of 23 ± 0.1 weeks after follow-up 1. Hematological parameters including hemoglobin, white blood cell (WBC) and platelet (PLT) counts, neutrophils, lymphocytes and creatinine were collected before treatment cycles and during follow-up examinations and changes between the different time points of laboratory analyses were noted as absolute and percentage changes. Common Terminology Criteria for Adverse Events version 5.0 (CTCAE v5.0) were used for grading hematological parameters. CTCAE grades for decreased PLT, neutrophil and lymphocyte counts, as well as anemia, are shown in Table 1. Patients with the same toxicity grading before and after PRRT, as well as patients who improved their grading in hematological toxicity, were excluded from this analysis. Measurements of tubular extraction rate (TER) resulted from ^99m^Tc-MAG3 renal scintigraphies performed prior to each cycle and after the end of treatment. To evaluate the development (recovery trend) of blood values in the further course after PRRT, hematological and renal parameters of patients in all four subgroups were frequently sampled from the referring endocrinologists and/or oncologist and were correlated with the time (weeks) after follow-up 2. These blood values were collected after a mean time after follow-up 2 of 41.7 ± 27.1 weeks in the Lutathera_8weeks_ group, 30.4 ± 19.7 weeks in the Lutathera_adapted_ group, 50.5 ± 29.2 weeks in the HA_8weeks_ group and 58.9 ± 41.1 weeks in the HA_adapted_ group.

### 2.5. Statistical Analysis

Data are reported as mean ± standard deviation, as stated. Demographics were compared between groups using Student’s *t*-test for metric variables and a Chi-squared test for non-metric data. Statistical comparison of absolute blood counts within all four groups was performed using Student’s *t*-test for normally distributed data (reported as mean) and a Mann–Whitney test for not normally distributed data (reported as median). Relative percentage changes were compared between the 4 groups using a two-way Anova test. *p*-values were adjusted to multiple comparisons using Tukey’s multiple comparison test. Percentage recovery of blood values was correlated with the time (weeks) after follow-up 2 using a Pearson’s correlation coefficient and easy linear regression. GraphPad Prism (version 8.4.3, GraphPad Software Inc., San Diego, CA, USA) was used for the statistical analysis and illustration of results. A significance level of *p* < 0.05 was applied in all analyses.

## 3. Results

### 3.1. Radiolabelling

Radiochemical purity was determined by radio-HPLC and ITLC and was always greater than 98%. Radio-TLC was carried out using ITLC-SG (SGI0001) strips (Agilent, Waldbronn, Germany) in 0.1 M citrate buffer (pH 5.0). More than 98% of the applied radioactivity appeared at R_f_ 0–0.1, representing the respective ^177^Lu-labeled peptide, whereas uncomplexed ^177^Lu (as Lu-citrate) appeared at R_f_ 0.9–1.0. Using a second radio-TLC system with ammonium acetate (77 g/L)/methanol (50/50 *v*/*v*) as mobile phase, the respective ^177^Lu-labeled peptides were detected at R_f_ 0.8–1.0, whereas ^177^Lu^3+^ or ^177^Lu-colloids appeared at R_f_ 0–0.1. The pH of the injected substance was between 4 and 6. Identity was tested using non-radioactive Lu-HA-DOTATATE by HPLC. The radioactive preparations were also tested according to Eur. Pharm for apyrogenicity and sterility and each preparation met the specifications.

### 3.2. Patients

A total number of 46 patients (*n* = 22 male; *n* = 24 female) with a mean age of 67 ± 0.7 years underwent all four PRRT cycles. Inclusion criteria for PRRT included patients with well-differentiated GEP-NETs (G1/2) with progression under ongoing SSI therapy and sufficient uptake on SSTR imaging. Primary tumor sites were the small intestine (ileum *n* = 20, jejunum *n* = 1, n.s. *n* = 3), rectum (*n* = 2), pancreas (*n* = 15) and stomach (*n* = 2). In three patients, the primary tumor site was not detectable (carcinoma of unknown primary, CUP). Metastatic locations included the liver (*n* = 43), the lymph nodes (*n* = 27), bone (*n* = 17), lung (*n* = 1) and peritoneal (*n* = 16) lesions. The majority of patients underwent surgery (*n* = 29) and biotherapy with somatostatin analogs (*n* = 32) before the administration of PRRT. Further treatments before PRRT included chemotherapy (capecitabine/temozolomide (CAPTEM) *n* = 6, streptozocin/5-fluorouracil *n* = 3, folinic acid/5-fluoruracil/oxaliplatin (FOLFOX) *n* = 1, gemcitabine *n* = 1) and everolimus (*n* = 1). Patient demographics are shown in Table 2. During four cycles of PRRT, the overall injected activity of all subgroups amounted to 29,066 MBq in the Lutathera_8weeks_ group, 29,068 MBq in the Lutathera_adapted_ group, 29,168 MBq in the HA_8weeks_ group and 29,612 MBq in the HA_adapted_ group. Significant differences in mean average activities applied to the four subgroups at every cycle of PRRT were detected at the second (Lutathera_8weeks_ 7262 vs. HA_8weeks_ 7388 MBq, *p* = 0.014; Lutathera_8weeks_ 7262 vs. HA_adapted_ 7403 MBq, *p* = 0.005), third (Lutathera_adapted_ 7243 vs. HA_adapted_ 7406 MBq, *p* = 0.018) and fourth (Lutathera_8weeks_ 7290 vs. HA_adapted_ 7436 MBq, *p* = 0.034) PRRT cycle.

### 3.3. Therapy Associated Toxicity During PRRT

#### 3.3.1. CTCAE Assessment

Table 3 shows the respective number of patients in each subgroup with CTCAE grading of anemia, thrombocytopenia, neutropenia and lymphocytopenia before and after therapy. Prior to PRRT, no severe subacute hematotoxicity (grade 3/4) was detected in any patient of the subgroups. After termination of PRRT, still no grade 3 or 4 anemia or neutropenia was registered in any of the treatment groups. The HA_adapted_ group showed one case (1/10, 10%) of grade 3 thrombocytopenia after therapy, whereas no patients with grade 3 or 4 thrombocytopenia were detected in the Lutathera_8weeks_, Lutathera_adapted_ and HA_8weeks_ group. Severe subacute lymphocytopenia (grade 3) was observed in 7/16 (44%) patients of the Lutathera_8weeks_ group, 3/10 (30%) patients of the Lutathera_adapted_ group, 2/10 (20%) patients of the HA_8weeks_ group and 8/10 (80%) patients of the HA_adapted_ group. Moreover, only one patient with grade 4 lymphocytopenia was detected in the Lutathera_adapted_ group (1/10, 10%). Patients who underwent chemotherapy in the past (*n* = 11) did not show higher rates of severe subacute hematotoxicity compared to patients without any chemotherapy pretreatment.

#### 3.3.2. Comparison of Absolute Blood Counts Before and After PRRT Within Each Subgroup

Table 4 shows absolute blood values of hemoglobin, PLT counts, WBC counts, neutrophil granulocytes and lymphocytes for all four therapy groups prior to compared to after PRRT (follow-up 2). In comparison to blood counts prior to PRRT, all subgroups showed a significant decrease in absolute PLT counts, WBC counts, neutrophil granulocyte counts and lymphocyte counts at follow-up 2. A significant reduction in absolute hemoglobin levels when comparing pre-PRRT values to values at follow-up 2 was detected in the Lutathera_8weeks_, HA_8weeks_ and HA_adapted_ subgroups. However, the Lutathera_adapted_ group showed no significant decrease in absolute hemoglobin levels after PRRT.

#### 3.3.3. Comparison of Percentage Changes in Hematological and Renal Parameters Between the Subgroups

##### Hemoglobin, Creatinine and TER

**Figure 1:**
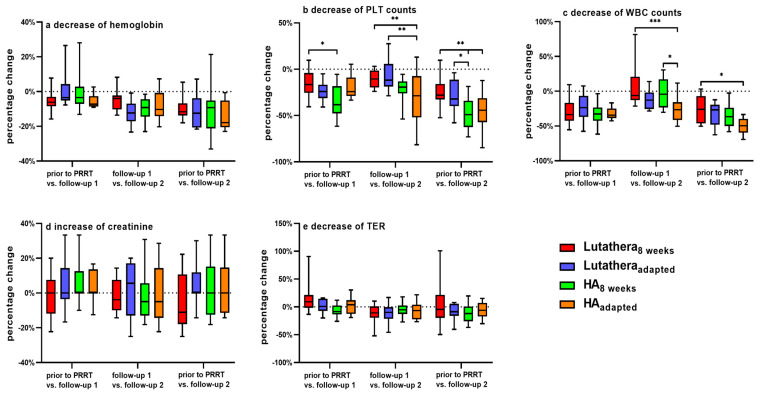
Changes (%) of (**a**) hemoglobin, (**b**) PLT count, (**c**) WBC count, (**d**) serum creatinine and (**e**) TER at three time points during PRRT compared between each subgroup. PLT count—platelet count. WBC count—white blood cell count. TER—tubular extraction rate. * = *p* ≤ 0.05. ** = *p* ≤ 0.01. *** = *p* ≤ 0.001.

Figure 1 shows percentage changes of hemoglobin, PLT counts, WBC counts, creatinine and TER compared between the subgroups over the course of PRRT. There were no significant differences in the percentage changes of hemoglobin, creatinine and TER between Lutathera_8weeks_ vs. Lutathera_adapted_, HA_8weeks_ vs. HA_adapted_, Lutathera_8weeks_ vs. HA_8weeks_, or Lutathera_adapted_ vs. HA_adapted_, respectively, when comparing values at baseline (prior to PRRT) with values at follow-up 1 and follow-up 2. Furthermore, there were no significant changes in between follow-up 1 and 2. Also, a cross-comparison between Lutathera_8weeks_ and Lutathera_adapted_ as well as HA_8weeks_ and HA_adapted_ showed no significant difference in hemoglobin, creatinine and TER values (*p* > 0.05, each) (Figure 1).

##### WBC Counts

WBC counts of the HA_adapted_ group decreased significantly in their percentage changes as follows: HA_adapted_ patients demonstrated a significantly higher decline (%) of WBC counts in between follow-up 1 and follow-up 2 when compared to HA_8weeks_ (HA_adapted_ = −25.2 ± 19 vs. HA_8weeks_ −2 ± 22.1 [%]; *p* = 0.032) but not when comparing WBC counts prior to PRRT and at follow-up 2 (HA_adapted_ = −49.9 ± 11.7 vs. HA_8weeks_ −35.4 ± 17.2 [%]; *p* = 0.31). HA_adapted_ patients also showed a significantly higher decline (%) of WBC counts in between follow up 1 and 2 (HA_adapted_ = −25.2 ± 19 vs. Lutathera_8weeks_ = +6.6 ± 27.4 [%]; *p* = 0.0003) and when comparing WBC counts prior to PRRT and at follow-up 2 to Lutathera_8weeks_ patients (HA_adapted_ = −49.9 ± 11.7 vs. Lutathera_8weeks_ = −26.3 ± 19.2 [%]; *p* = 0.011) (Figure 1).

There were no significant differences in percentage changes in WBC counts between Lutathera_8weeks_ vs. Lutathera_adapted_, Lutathera_adapted_ vs. HA_adapted_ and Lutathera_8weeks_ vs. HA_8weeks_, respectively.

##### PLT Counts

PLT counts decreased significantly as follows:

- HA_8weeks_: HA_8weeks_ patients presented a significantly higher reduction (%) of PLT counts at follow-up 1 in contrast to values prior to PRRT when compared to the Lutathera_8weeks_ group (HA_8weeks_ = −34.7 ± 16.9 vs. Lutathera_8weeks_ = −15.1 ± 14.5 [%]; *p* = 0.023). Moreover, patients of the HA_8weeks_ group also showed a significantly higher decline (%) of PLT counts at follow-up 2 in comparison to values prior to PRRT when compared to Lutathera_8weeks_ patients (HA_8weeks_ = −48.3 ± 16.9 vs. Lutathera_8weeks_ = −24.1 ± 15.1 [%]; *p* = 0.003) as well as when compared to patients of Lutathera_adapted_ (HA_8weeks_ = −48.3 ± 16.9 vs. Lutathera_adapted_ = −28.3 ± 17.2 [%]; *p* = 0.042) (Figure 1).
- HA_adapted_: A significantly higher decline (%) of PLT counts in between follow-up 1 and follow-up 2 was measured in HA_adapted_ patients in comparison to Lutathera_adapted_ patients (HA_adapted_ = −32.1 ± 30.2 vs. Lutathera_adapted_ = −6.8 ± 17.1 [%], *p* = 0.005) as well as in comparison to Lutathera_8weeks_ patients (HA_adapted_ = −32.1 ± 30.2 vs. Lutathera_8weeks_ = −15.1 ± 14.5 [%], *p* = 0.01). HA_adapted_ patients also demonstrated a significantly higher decline (%) of PLT counts at follow-up 2 (HA_adapted_ = −46.1 ± 22.9 vs. Lutathera_8weeks_ = −24.1 ± 15.1 [%], *p* = 0.008) when compared to baseline values of Lutathera_8weeks_ patients (Figure 1).

#### 3.3.4. Recovery Trends of Hematological and Renal Parameters of All Therapy Subgroups

**Figure 2:**
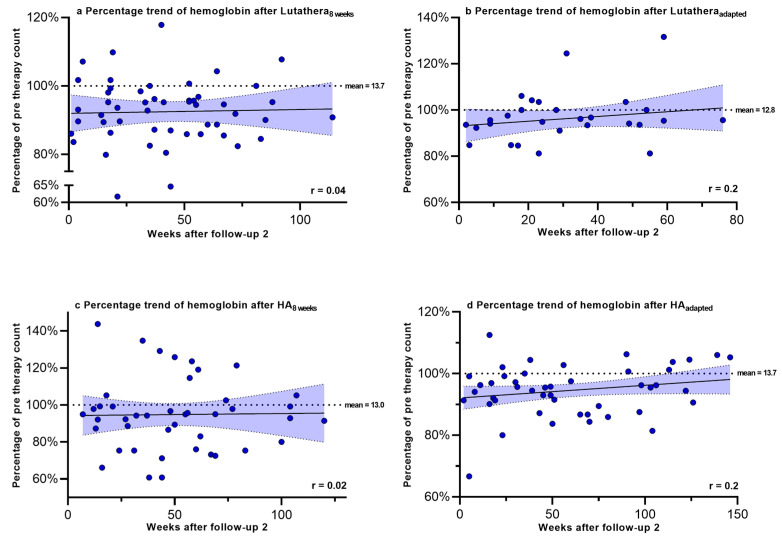
Hemoglobin levels expressed as percentages of values prior to PRRT and correlated with weeks after follow-up 2. Blut dots mark individual hemoglobin values measured in the patients of the different subgroups in the following weeks after PRRT. The dotted line shows the mean hemoglobin value prior to PRRT.

**Figure 3:**
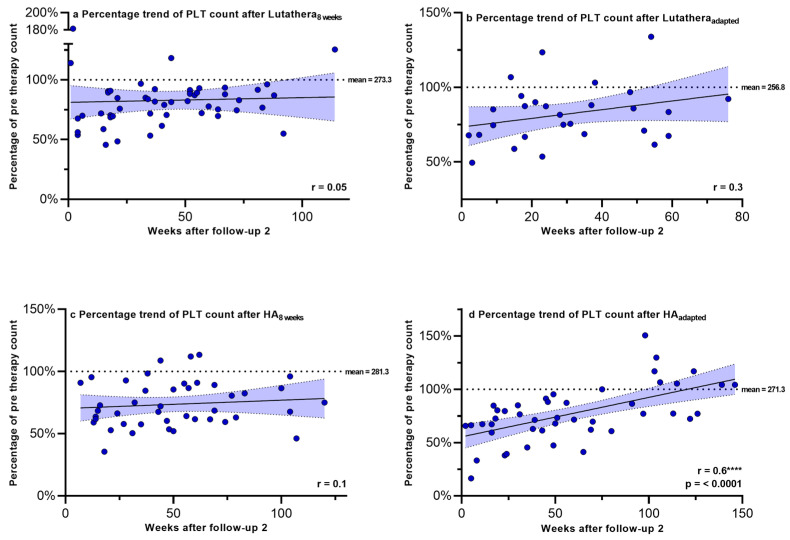
Platelet counts expressed as percentages of values prior to PRRT and correlated with weeks after follow-up 2. Blut dots mark individual PLT counts measured in the patients of the different subgroups in the following weeks after PRRT. The dotted line shows the mean PLT counts prior to PRRT. PLT count–platelet count. **** = *p* ≤ 0.0001.

**Figure 4:**
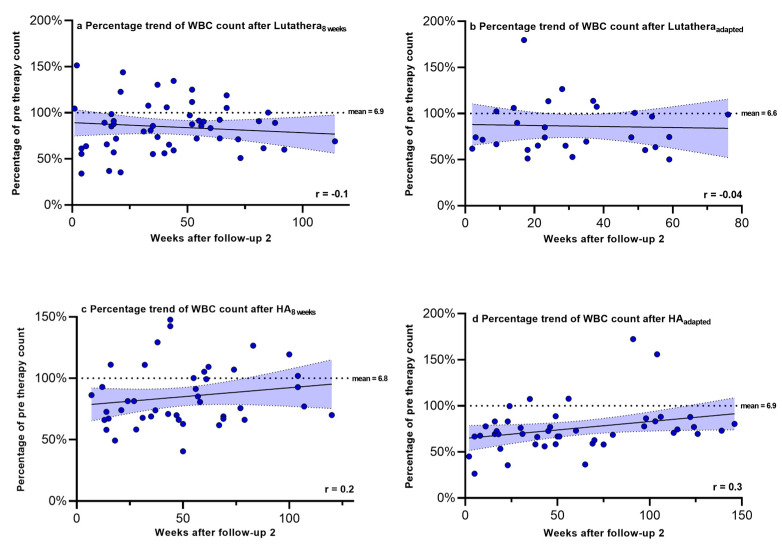
White blood cell counts expressed as percentages of values prior to PRRT and correlated with weeks after follow-up 2. Blut dots mark individual WBC counts masured in the patients of the different subgroups in the following weeks after PRRT. The dotted line shows the mean WBC counts prior to PRRT. WBC count—white blood cell count.

**Figure 5:**
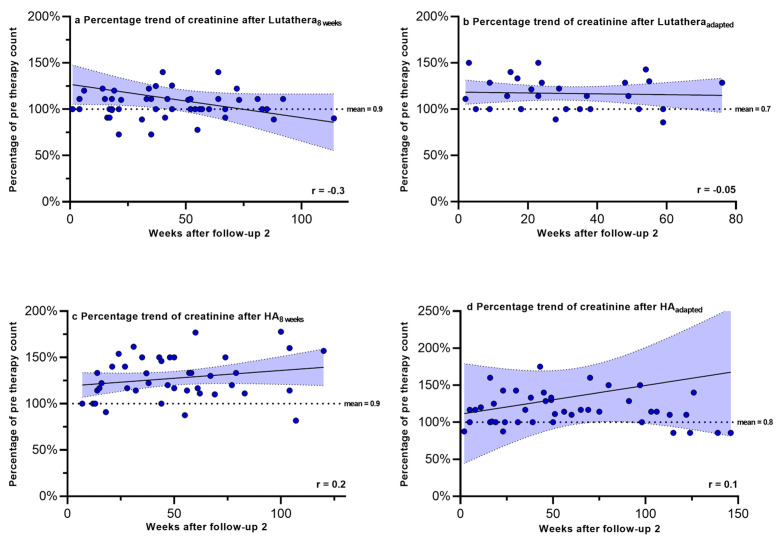
Creatinine expressed as percentages of values prior to PRRT and correlated with weeks after follow-up 2. Blut dots mark individual creatinine values masured in the patients of the different subgroups in the following weeks after PRRT. The dotted line shows the mean creatinine values prior to PRRT.

Hemoglobin, PLT counts, WBC counts and creatinine were regularly measured after PRRT in order to monitor recovery trends up to a mean time after follow-up 2 of 58.9 weeks. Figure 2, Figure 3, Figure 4 and Figure 5 show the progression of these hematological and renal parameters over the weeks after follow-up 2. These parameters are expressed as percentage changes compared to values prior to PRRT. HA_adapted_ patients demonstrated a significant positive correlation between the level of PLT counts and the time (in weeks) after follow-up 2 (r = 0.6, *p* < 0.0001).

No significant correlations in the recovery trends of PLT counts, hemoglobin, WBC counts and creatinine after follow-up 2 could be detected in any of the other subgroups.

## 4. Discussion

In this study, we compared the hemato- and nephrotoxicity of PRRT with both locally manufactured ^177^Lu-HA-DOTATATE and Lutathera^®^ and investigated the potential influence of different time intervals between PRRT cycles in patients with GEP-NETs G1/G2.

Previous studies showed that approximately 5–15% of patients develop subacute hematological toxicity; Bergsma et al. investigated 320 NET patients treated with PRRT with ^177^Lu-DOTATATE. Severe subacute hematotoxicity (grade 3/4) was found in 34/200 (11%) patients, including thrombocytopenia in 25 (8%), leukocytopenia in 17 (5%), anemia in 10 (3%) and pancytopenia (1%) [14]. A similar occurrence of subacute hematological toxicity (grade 3/4) was discovered by de Vries-Huizing et al. in 8/100 (8%) patients, while mild/moderate hematotoxicity (grade 1/2) was seen in 38/100 (38%) patients [20]. In the prospective observational study of 200 NET patients treated with ^177^Lu-DOTATATE PRRT by Garske-Román et al., 30/200 patients (15%) developed grade 3 or 4 hematotoxicity [21].

These findings are in line with our study results; there were no grade 3 or 4 anemia or neutropenia detected in patients treated with ^177^Lu-HA-DOTATATE and Lutathera^®^. Only one case of severe subacute thrombocytopenia (grade 3) was detected in the HA_adapted_ group (1/10, 10%), whereas no grade 3 thrombocytopenia was found in patients treated with Lutathera^®^ or in the HA_8weeks_ group. However, more incidents of grade 3 lymphocytopenia were detected: 7/16 (44%) patients of the Lutathera_8weeks_ group, 3/10 (30%) patients of the Lutathera_adapted_ group, 2/10 (20%) patients of the HA_8weeks_ group and 8/10 (80%) patients of the HA_adapted_ group showed severe subacute lymphocytopenia. Only one patient with grade 4 lymphocytopenia was detected in the Lutathera_adapted_ group (1/10, 10%).

Absolute and percentage changes in kidney function (creatinine, TER) remained constant during PRRT in all subgroups and no nephrotoxicity within the observation time was detected in any subgroup which is in line with previous studies that also unveiled low incidents of nephrotoxicity. Garske-Román et al. described grade 1 nephrotoxicity in 38/200 (19%) and grade 2 nephrotoxicity in only 8/200 (4%) patients [21].

All four subgroups showed a significant decrease in absolute blood values for hemoglobin, PLT counts, WBC counts, neutrophil granulocytes and lymphocytes between, prior to and after PRRT (*p* < 0.05, each), except for the absolute hemoglobin levels of the Lutathera_adapted_ group. Regarding percentage changes in laboratory parameters, only patients of the HA_adapted_ and HA_8weeks_ group had a significant decrease in WBC and PLT counts during the therapy course and/or after PRRT compared to the other subgroups. There was no significant percentage degradation of any other hematological or renal parameter (hemoglobin, creatinine, TER) upon comparison between the subgroups. Only patients with longer treatment intervals under ^177^Lu-HA-DOTATATE (HA_adapted_) showed a statistically significant correlation regarding long-term recovery of PLT counts, while all the other hematological and renal parameters showed no significant correlation in this longer follow-up time in any of the subgroups. These results suggest that patients who underwent treatment with locally manufactured ^177^Lu-HA-DOTATATE might be at higher risk of developing significant changes in their PLT and WBC counts directly after PRRT compared to patients who underwent PRRT with Lutathera^®^, independently of the treatment intervals. However, patients who were treated with locally manufactured ^177^Lu-HA-DOTATATE PRRT in intervals of 11 weeks might be the subgroup that has a better long-term recovery of PLT counts after termination of PRRT compared to the other subgroups. That said, it should be emphasized that an 11-week dose interval should only be considered if tolerance to treatment is not associated with decreased treatment efficacy.

This overall low to moderate incidence of significant changes in hematological parameters directly after PRRT is in line with previous studies that could demonstrate a low absorbed dose of less than 0.2 Gy per treatment cycle of 7.4 GBq [22] even if individual risk factors of patients might have a relevant impact on the prediction of toxicity [14]. Overall, the 11 patients of our study, who received chemotherapy prior to PRRT, showed similar rates of severe subacute hematotoxicity (grade 3) in comparison to patients that were not treated with chemotherapy beforehand. That suggests that pre-treatment with chemotherapy prior to PRRT is safe and not necessarily associated with hematotoxicity in the observational period we have included in this study, even if the number of patients with prior chemotherapy is small. Our findings support a previous paper by Fröss-Baron et al., who reported hematotoxicity following PRRT being not related to previous chemotherapy regimens in 102 patients with advanced pancreatic NETs [23]. Given the increasing frequency of the use of PRRT also for other somatostatin receptor-expressing tumor entities such as lung carcinoids, pheochromocytoma/paraganglioma (PPGLs) and meningiomas, the results of our study motivate that also the use of locally manufactured ^177^Lu-HA-DOTATATE can be a safe treatment option without too many hematotoxic side effects within the first year after therapy.

A limiting factor of this study is the relatively small cohort size and the heterogeneity of pre-treatments. In addition, for assessing recovery trends of hematological parameters, the collection of blood values after the termination of PRRT was not uniform (range: 30.4–58.9 weeks after follow-up 2), while long-term follow-up data that are extending the time span of more than one year are currently missing. Given the risk of developing hematological diseases such as myelodysplastic syndrome or acute myeloid leukemia years after PRRT, longer observational periods than that performed in this study are crucial [6,24,25,26].

## 5. Conclusions

Comparing the hematotoxicity in patients that were treated with locally manufactured ^177^Lu-HA-DOTATATE to patients that were treated with Lutathera^®^ and assessing different treatment intervals in both groups (8 vs. 11 weeks) revealed that there is overall a low to moderate incidence of significant changes in hematological and renal parameters within the first year after therapy. These changes primarily relate to a significant percentage decrease in WBC and PLT counts in patients treated with locally manufactured ^177^Lu-HA-DOTATATE directly after four cycles of PRRT. Recovery trends of hematological and renal parameters up to one year after PRRT suggest that patients treated with locally manufactured ^177^Lu-HA-DOTATATE might benefit from a longer treatment interval of 11 weeks regarding their PLT counts. Prospective trials including longer observational periods than performed in this study are needed to assess the impact of PRRT-induced hematotoxicities to develop diseases such as therapy-related myeloid neoplasms [27].

## Figures and Tables

**Table 1:** Common Terminology Criteria for Adverse Events version 5.0 (CTCAE v5.0) for decreased platelet (PLT), neutrophil and lymphocyte counts and anemia. LLN—lower limit of normal.

	Grade 1	Grade 2	Grade 3	Grade 4
PLT count decreased	<LLN to 75.000/µL	50.000–75.000/µL	25.000–50.000/µL	<25.000/µL
Anemia	<LLN to 10 g/dL	8.0–10.0 g/dL	<8.0 g/dL	life-threatening consequences
Neutrophil count decreased	<LLN to 1.500/µL	1.000–1.500/µL	500–1.000/µL	<500/µL
Lymphocyte count decreased	<LLN to 800/µL	500–800/µL	200–500/µL	<200/µL

**Table 2:** Patient characteristics. ♂—male. ♀—female.

(Mean ± SD)	All	Lutathera_8weeks_	Lutathera_adapted_	HA_8weeks_	HA_adapted_	*p*-Value
*n*	46	16	10	10	10	
Sex	♂ 22 ♀ 24	♂ 10 ♀ 6	♂ 3 ♀ 7	♂ 5 ♀ 5	♂ 4 ♀ 6	0.426
Age [y]	66.5 ± 0.7	67.5 ± 12	65.9 ± 11	66.1 ± 13	66.6 ± 11	0.985
Time since initial diagnosis [m]	47.6 ± 16	64.1 ± 66	57.5 ± 42	40.5 ± 48	28.3 ± 34	0.337
Ki-67 [%]	7.3 ± 1.6	7 ± 6	5.7 ± 3	9.6 ± 5	7 ± 6	0.431
Grading	G1 *n* = 11, G2 *n* = 35	G1 *n* = 4, G2 *n* = 12	G1 *n* = 3, G2 *n* = 7	G1 *n* = 2, G2 *n* = 8	G1 *n* = 2, G2 *n* = 8	0.944

**Table 3:** Number of patients in all therapy subgroups with evidence of hematological toxicity according to CTCAE v5.0 criteria prior to and after termination of PRRT.

	Anemia			Thrombocytopenia			Neutropenia			Lymphocytopenia			
CTCAE grade	1	2	3	1	2	3	1	2	3	1	2	3	4
**Prior to PRRT**													
Lutathera_8weeks_	-	-	-	-	-	-	-	-	-	4	-	-	-
Lutathera_adapted_	1	-	-	-	-	-	1	-	-	3	1	-	-
HA_8weeks_	1	-	-	-	-	-	-	-	-	2	-	-	-
HA_adapted_	-	-	-	-	-	-	-	-	-	1	1	-	-
**After 4 cycles of PRRT**													
Lutathera_8weeks_	8	-	-	4	-	-	1	-	-	1	1	7	-
Lutathera_adapted_	4	2	-	3	-	-	1	1	-	-	3	3	1
HA_8weeks_	2	1	-	7	-	-	-	1	-	2	6	2	-
HA_adapted_	3	1	-	4	1	1	1	1	-	2	-	8	-

**Table 4:** Absolute blood counts of hemoglobin, PLT, WBC, neutrophil granulocytes and lymphocytes as well as creatinine and total-TER within each subgroup before and after PRRT, with corresponding *p*-values.

	Lutathera_8weeks_ Prior to PRRT vs. Lutathera_8weeks_ After PRRT	Lutathera_adapted_ Prior to PRRT vs. Lutathera_adapted_ After PRRT	HA_8weeks_ Prior to PRRT vs. HA_8weeks_ After PRRT	HA_adapted_ Prior to PRRT vs. HA_adapted_ After PRRT
Hemoglobin [mg/dL]	13.73 vs. 12.36, *p* = 0.011	12.75 vs. 11.27, *p* = 0.062	13.8 vs. 11.25, *p* = 0.02	13.66 vs. 11.82, *p* = 0.041
PLT counts [G/L]	273.3 vs. 202.8, *p* = 0.024	256.8 vs. 181, *p* = 0.017	281.3 vs. 143.5, *p* ≤ 0.0001	271.3 vs. 139.6, *p* = 0.001
WBC counts [G/L]	7.0 vs. 4.79, *p* = 0.002	6.63 vs. 4.32, *p* = 0.021	6.78 vs. 4.25, *p* = 0.001	6.93 vs. 3.43, *p* ≤ 0.0001
Neutrophile granulocytes [G/L]	4.61 vs. 3.45, *p* = 0.01	4.48 vs. 3.0, *p* = 0.027	4.45 vs. 2.89, *p* = 0.009	4.4 vs. 2.44, *p* = 0.001
Lymphocytes [G/L]	1.38 vs. 0.54, *p* = 0.02	1.21 vs. 0.57, *p* = 0.01	1.54 vs. 0.68, *p* ≤ 0.0001	1.81 vs. 0.43, *p* ≤ 0.0001
Creatinine [mg/dL]	0.9 vs. 0.87, *p* = 0.36	0.77 vs. 0.82, *p* = 0.566	0.85 vs. 0.85, *p* = 0.989	0.79 vs. 0.8, *p* = 0.881
Total-TER [mL/min]	201.9 vs. 191.3, *p* = 0.627	217.5 vs. 210.5, *p* = 0.288	211.4 vs. 189, *p* = 0.357	228.4 vs. 210.7, *p* = 0.365

## Data Availability

The data presented in this study are available on request from the corresponding author due to specific reasons.
